# Protein structure refinement using a quantum mechanics-based chemical shielding predictor†
†Electronic supplementary information (ESI) available. See DOI: 10.1039/c6sc04344e



**DOI:** 10.1039/c6sc04344e

**Published:** 2016-12-01

**Authors:** Lars A. Bratholm, Jan H. Jensen

**Affiliations:** a Department of Chemistry , University of Copenhagen , Copenhagen , Denmark . Email: larsbratholm@gmail.com ; Email: jhjensen@chem.ku.dk ; http://www.twitter.com/janhjensen

## Abstract

The accurate prediction of protein chemical shifts using a quantum mechanics (QM)-based method has been the subject of intense research for more than 20 years but so far empirical methods for chemical shift prediction have proven more accurate. In this paper we show that a QM-based predictor of a protein backbone and CB chemical shifts (ProCS15, *PeerJ*, 2016, 3, e1344) is of comparable accuracy to empirical chemical shift predictors after chemical shift-based structural refinement that removes small structural errors. We present a method by which quantum chemistry based predictions of isotropic chemical shielding values (ProCS15) can be used to refine protein structures using Markov Chain Monte Carlo (MCMC) simulations, relating the chemical shielding values to the experimental chemical shifts probabilistically. Two kinds of MCMC structural refinement simulations were performed using force field geometry optimized X-ray structures as starting points: simulated annealing of the starting structure and constant temperature MCMC simulation followed by simulated annealing of a representative ensemble structure. Annealing of the CHARMM structure changes the CA-RMSD by an average of 0.4 Å but lowers the chemical shift RMSD by 1.0 and 0.7 ppm for CA and N. Conformational averaging has a relatively small effect (0.1–0.2 ppm) on the overall agreement with carbon chemical shifts but lowers the error for nitrogen chemical shifts by 0.4 ppm. If an amino acid specific offset is included the ProCS15 predicted chemical shifts have RMSD values relative to experiments that are comparable to popular empirical chemical shift predictors. The annealed representative ensemble structures differ in CA-RMSD relative to the initial structures by an average of 2.0 Å, with >2.0 Å difference for six proteins. In four of the cases, the largest structural differences arise in structurally flexible regions of the protein as determined by NMR, and in the remaining two cases, the large structural change may be due to force field deficiencies. The overall accuracy of the empirical methods are slightly improved by annealing the CHARMM structure with ProCS15, which may suggest that the minor structural changes introduced by ProCS15-based annealing improves the accuracy of the protein structures. Having established that QM-based chemical shift prediction can deliver the same accuracy as empirical shift predictors we hope this can help increase the accuracy of related approaches such as QM/MM or linear scaling approaches or interpreting protein structural dynamics from QM-derived chemical shift.

## Abstract 2

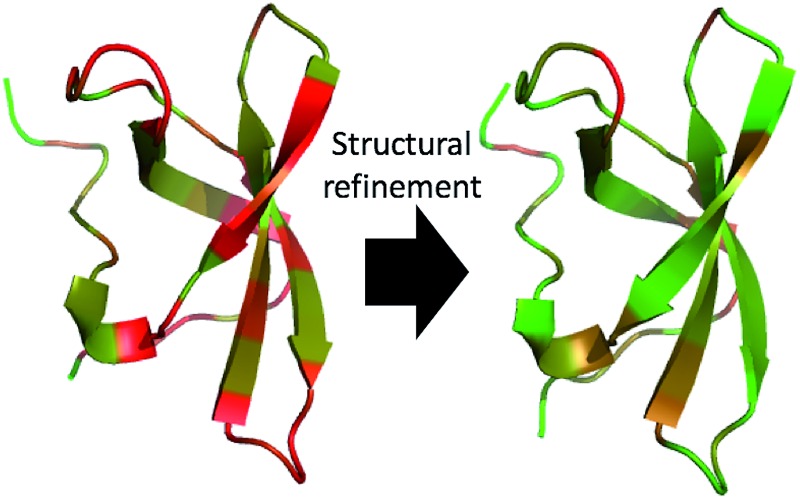
We show that a QM-based predictor of a protein backbone and CB chemical shifts is of comparable accuracy to empirical chemical shift predictors after chemical shift-based structural refinement that removes small structural errors (errors in chemical shifts shown in red).

## Introduction

Chemical shifts are very sensitive to the molecular structure and computational methods that can accurately predict chemical shifts from structure (and *vice versa*) are valuable tools for protein structure determination and validation. These methods, *e.g.* CamShift,^1^ PPM_One,^2^ Sparta+,^3^ shAIC,^4^ and ShiftX2,^5^ are typically based on approximate physical models with adjustable parameters that are optimized by minimizing the discrepancy between experimental and predicted chemical shifts computed using protein structures derived from X-ray crystallography. Alternatively, protein chemical shifts can be predicted using computational quantum mechanics (QM), either indirectly using QM-derived models such as SHIFTS,^6,7^ CheShift,^8,9^ and ProCS,^10,11^ or directly using QM/MM or linear scaling approaches.^12–15^

In principle the QM-based methods offer several advantages over the empirical methods. As case^16^ notes: “Quantum models allow the study of unusual conformations, including fibrils, partially disordered systems, or other unusual configurations that might not be represented in the existing databases of shifts. They can take account of the effects of ligands or cofactors, and can be applied to carbohydrates, nucleic acids, and other biochemical entities”. Furthermore, they should be more appropriate for validating structural ensembles “since we know exactly what structures (or structural ensemble) are involved, avoiding the ‘structural noise’ that arises in the empirical models from the fact that the structural ensemble leading to the observed shifts is not known”. However, QM-based methods tend to yield chemical shifts that, on average, are less accurate than the empirical methods.

Two main reasons for the lower accuracy have been advanced: 1) the QM-based predictors are more sensitive to small structural errors than empirical predictors and 2) lack of dynamical averaging which is implicitly included in the empirical methods. For example, Vila *et al.* ^8^ showed that CA chemical shifts predicted by CheShift were able to better discriminate between decoy structures from native conformations than SHIFTS and SHIFTX (although SPARTA performed equally well) and He *et al.* ^17^ showed that the ensemble average of proton chemical shifts predicted by their linear scaling AF-QM method improves the correlation with experimental results (although SHIFTS and SHIFTX predictions remain more accurate).

Despite >20 years of work^18^ no study so far has demonstrated that QM-based protein chemical shift predictions can deliver accuracies similar to empirical methods for a variety of protein structures and atom types, an important and necessary first step if their advantages (rigor, generality, *etc.*) are to be realized and embraced. In this paper we perform chemical shift-based structural refinement of 17 proteins to demonstrate that the primary source of error for QM-based chemical predictions are small structural errors and that a QM-based method can predict a protein backbone and CB chemical shifts with accuracies very similar to empirical methods once these errors are removed.

## Theory

### Overview

Markov Chain Monte Carlo (MCMC) simulations are performed with a hybrid energy function based on a standard force field energy (*E*_FF_) augmented by an energy term (*E*_CS_) that reflects the agreement between predicted and experimental chemical shifts

1 *E*_hybrid_ = *E*_FF_ + *wE*_CS_

The optimum weight (*w*) of the chemical shift data is determined probabilistically as part of the simulation as described below. If one assumes that the predicted chemical shifts follows a normal distribution (*p*_CS_) around the experimental values,^19^eqn (1) can be rewritten as2
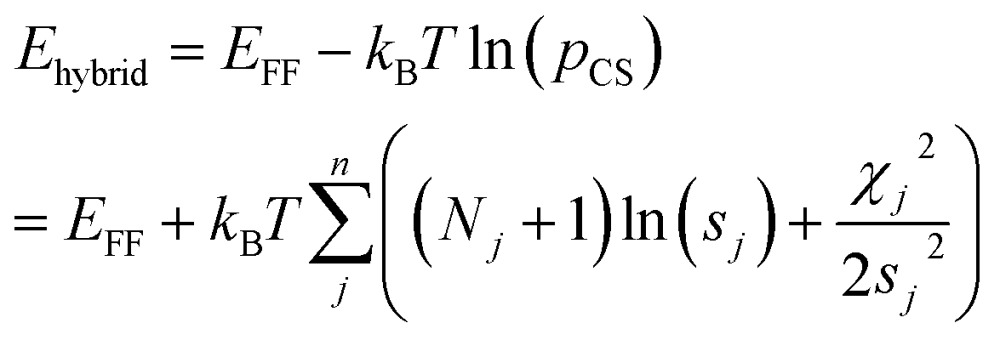
here *k*_B_ is Boltzmann's constant, *T* is the temperature of the simulation, *n* is the number of different atom types (C_α_, C_β_, H_α_, *etc.*) for which chemical shifts are available, *N*_*j*_ is the number of chemical shifts of nuclei type *j*, and *s*_*j*_ is the standard deviation in the prediction of chemical shift-type *j*. Finally, *χ*_*j*_^2^ is defined as3
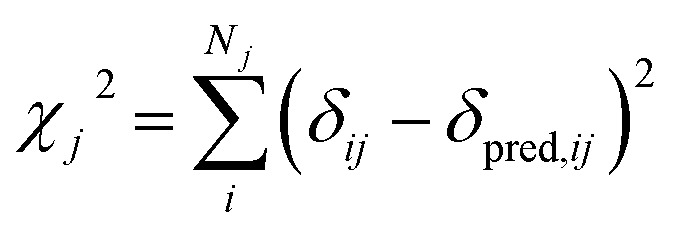
where *δ*_*ij*_ is the experimental chemical shift for nucleus *i* of type *j* and *δ*_pred,*ij*_ is the corresponding predicted value. From this it is seen that the standard deviations are effectively describing the weight *w* of the experimental data.

In this study we use ProCS15 (ref. 11) to predict the corresponding isotropic chemical shielding value *σ*_*i*_, which we relate to *δ*_*ij*_ by

4 *δ*_pred,*ij*_ = *a*_*j*_*σ*_*ij*_ + *b*_*j*_

The slope, *a*_*j*_, and offset, *b*_*j*_, are determined by the agreement between the predicted and experimental chemical shifts for each atom type (see next subsection).

### Details

Following the inferential structure determination approach by Rieping, Habeck and Nilges,^20^ the hybrid energy corresponds to the joint posterior density for all unknown parameters

5 *p*(*X*,***θ***|{***δ***}) ∝ *p*({***δ***}|*X*,***θ***)*π*(*X*)*π*(***θ***)

6 *E*_hybrid_ = –*k*_B_*T* ln(*p*(*X*,***θ***|{***δ***}))

7 *E*_CS_ = –*k*_B_*T* ln(*p*({***δ***}|*X*,***θ***)*π*(***θ***))

where boldface is used to represent vectors over different atom types, {·} is used to represent the set of all residues, {***δ***} is the experimental data, *X* the given structure and ***θ*** unknown model parameters (***a***,***b***,***s***).

Here a Bayesian linear regression model is used to describe the agreement between the prediction of isotropic chemical shieldings by ProCS15, {***σ***}, and the chemical shifts found experimentally, {***δ***}, such that ***δ***_*i*_ = ***a***·***σ***_***i***_ + ***b*** + ***ε*** for residue *i*, with ***ε*** being a zero centered normal error with a diagonal covariance matrix **Σ** = ***s*** ^T^·**I**·***s***, with *I* being the identity matrix. Thus8
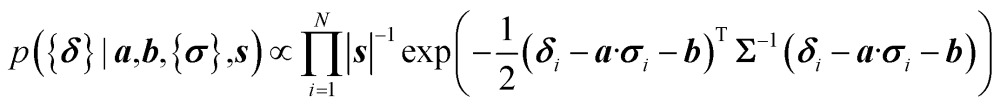

9
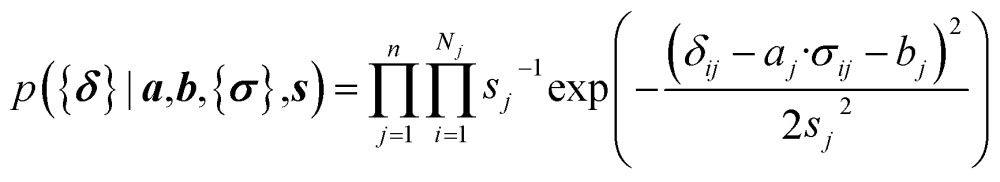



Non-informative priors are used for the model parameters:^21–23^
10
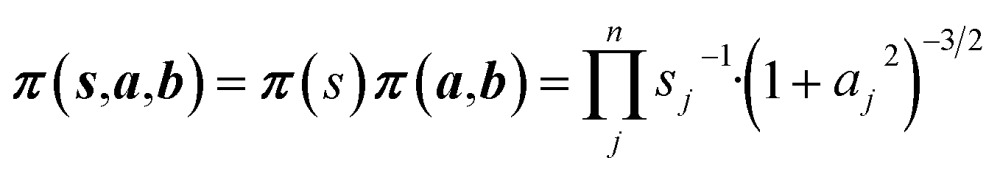



The parameters ***a*** and ***b*** are marginalized out using Laplace's method:11
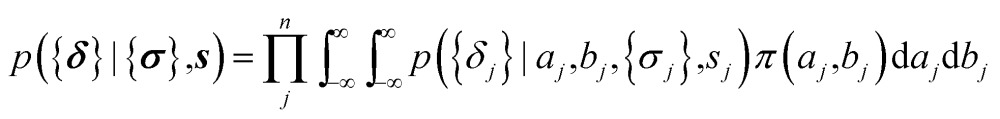

12
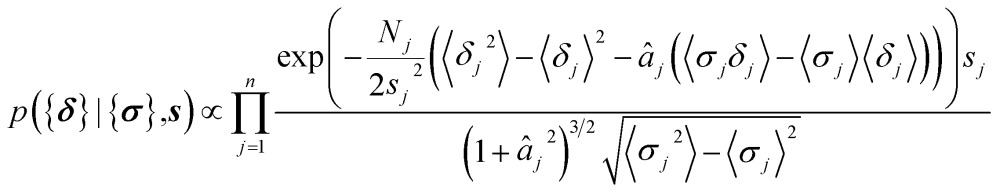
with *â*_*j*_ being13
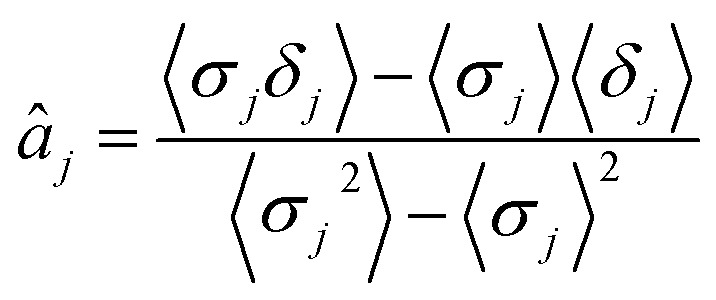
and · denoting the mean.

## Computational methodology

Seventeen protein X-ray structures are used in this study which are listed in Table S1.† In several cases the sequences of the proteins for which X-ray structures and chemical shifts are available differ slightly from those for which chemical shifts were measured and the corresponding residues were changed in the X-ray structure using FoldX 4.^24^ Additionally some side chain coordinates in the PDB files are missing and were added in a similar fashion. The structures were energy minimized with the CHARMM22/CMAP force field^25^ and the GB/SA solvation model^26^ implemented in TINKER^27^ with a convergence criterion of 0.01 kcal mol^–1^ Å^–1^.

The CHARMM/CMAP energy minimized structures were used as starting points for two different kinds of Markov Chain Monte Carlo (MCMC) simulations, carried out using PHAISTOS^28^ with the Metropolis–Hastings acceptance criterion^29^ using the hybrid energy function described above: simulated annealing, and constant temperature simulations. The physical force field CHARMM36, with the EEF1-SB solvent model, were used in the simulations.^30^ The simulated annealing protocol consisted of simply lowering the temperature from *t*_start_ = 300 K to *t*_end_ = 3 K over *N*_steps_ = 10 M steps, with the temperature at step *i* being 
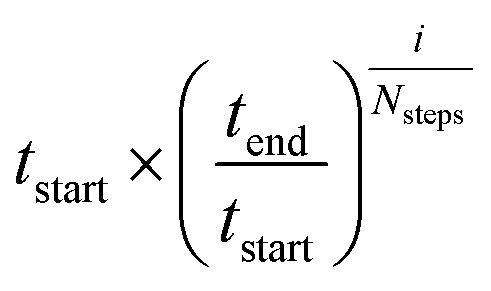
. In the constant temperature (300 K) MCMC simulations, the resulting set of structures do not represent a thermodynamic ensemble because a hybrid energy function is used. For the simulated annealing simulations, four independent Metropolis–Hastings simulations were performed for each protein, for 10 million (M) MC steps (40 M in total). For the ensembles, eight threads are performed for each protein for 50 M steps (400 M in total). The conformational degrees of freedom explored in the simulations were restricted to the backbone and side-chain dihedral angles (*φ*, *ψ*, *χ*) as well as the backbone bond angles. The physical move set was comprised of 20% uniform and 30% local single side chain moves, 40% CRISP local backbone dihedral angle moves^31^ and 10% CRA backbone bond angle moves.^32^ An additional 5% moves were added to sample the standard deviation in the chemical shift energy term.^19^ The ClusCo program was used to extract a representative structure from the ensemble using hierarchical agglomerative clustering.

CheShift-2 (ref. 8 and 9) calculations were performed with the CheShift-2 PyMOL-plugin^33^ found at ; http://github.com/aloctavodia/cheshift. CamShift,^1^ PPM One,^2^ Sparta+,^3^ shAIC,^4^ and ShiftX2 (ref. 5) calculations are performed using the stand-alone predictors. Scripts to automate these predictions can be found at ; http://github.com/larsbratholm/cs_prediction.

Much of the variation in some of the chemical shifts comes from the nature of the side-chain itself and the neighboring side chains which can lead to inflated *r*-values. To separate the contributions of the sequence and the structure we subtract the measured sequence corrected random coil values^34^ from all predicted and experimental values. Note that this does not affect the computed RMSD values.

Outliers in the predicted chemical shifts are identified using the generalized extreme studentized deviate test^35^ and removed before computing RMSD, *r* values, and slopes.

## Results and discussion

### Chemical shift prediction using CHARMM minimized X-ray structures

The first row of Table 1 lists average RMSD and *r* values relative to experiments for chemical shift values computed using ProCS15 for 17 protein structures minimized using the CHARMM/CMAP force field. The RMSD values are generally very similar to those computed previously for ubiquitin and protein GB3,^11^ with the exception of N, where the average RMSD is 0.5 to 0.8 ppm lower. The *r* values for CA and CB are also quite similar to those obtained previously, but are significantly lower for the remaining nuclei.

**Table 1:** Average RMSD (in ppm) and *r* values relative to experiments of chemical shifts computed using 17 CHARMM minimized protein structures and several chemical shift predictors

	CA	CB	C	HA	H	N
ProCS15	1.6 (0.71)	1.9 (0.44)	1.7 (0.36)	0.30 (0.76)	0.55 (0.51)	3.4 (0.58)
CheShift-2	1.5 (0.65)	1.7 (0.48)				
CamShift	1.1 (0.80)	1.2 (0.72)	1.2 (0.73)	0.27 (0.81)	0.49 (0.69)	3.0 (0.63)
PPM_One	0.8 (0.90)	1.0 (0.83)	1.0 (0.78)	0.21 (0.90)	0.41 (0.78)	2.2 (0.80)
Sparta+	0.8 (0.91)	1.0 (0.83)	0.9 (0.82)	0.23 (0.87)	0.41 (0.77)	2.2 (0.81)
shAIC	0.8 (0.90)	1.0 (0.83)	0.9 (0.83)	0.21 (0.89)	0.42 (0.75)	2.2 (0.81)
ShiftX2	0.6 (0.92)	0.7 (0.88)	0.7 (0.88)	0.16 (0.92)	0.31 (0.85)	1.8 (0.84)

As we observed previously^11^ the RMSD values for ProCS15 are significantly higher (0.5–1.4 ppm for carbon and N atoms) than those for commonly used empirical chemical shift predictors and very similar to CheShift values, while the corresponding ProCS15 *r* values are lower than for the empirical methods and similar to CheShift. We now show that the agreement with experiment can be significantly improved for ProCS15 by making relative small changes to the protein structure.

### Chemical shift based structural refinement using ProCS15

The second row of Table 2 (labeled “Annealed CHARMM”) lists the average RMSD and *r* values computed using the lowest energy structures obtained by minimizing the hybrid energy function described in the Theory section, starting from the CHARMM/CMAP minimized structures using simulated annealing as described in the Computational methodology section. The data indicate that such an annealing lowers the RMSD values relative to using CHARMM/CMAP structures (labeled “CHARMM”) by between 0.2–0.8 ppm for carbon and nitrogen and by 0.12 and 0.16 ppm for HA and H. The improvements in RMSD are largest for CA and N, and more modest for CB and C. While the RMSD-lowering is relatively small for HA and H, the *r* values are increased by 0.21 and 0.25, respectively. The *r* values for the remaining atoms are also increased, by between 0.11 and 0.20.

**Table 2:** Average RMSD (in ppm) and *r* value relative experiments of chemical shifts computed using ProCS15 for 17 different proteins and various structural refinement techniques (explained in the text)

	CA	CB	C	HA	H	N
CHARMM	1.6 (0.71)	1.9 (0.44)	1.7 (0.36)	0.30 (0.76)	0.55 (0.51)	3.4 (0.58)
Annealed CHARMM	0.8 (0.91)	1.5 (0.59)	1.5 (0.46)	0.19 (0.91)	0.39 (0.78)	2.8 (0.72)
Ensemble average	0.6 (0.94)	1.4 (0.64)	1.4 (0.55)	0.19 (0.92)	0.42 (0.76)	2.3 (0.80)
Annealed ensemble	0.6 (0.95)	1.5 (0.60)	1.5 (0.53)	0.17 (0.92)	0.40 (0.78)	2.6 (0.75)

In order to explore a larger region of phase space and the effect of conformational averaging we perform a constant temperature Monte Carlo simulation for each protein using the hybrid energy function. The resulting structures are used to compute an average chemical shielding value for each nucleus, which is then related to the chemical shift by linear regression against the experimental values for each protein. The RMSD from the experimental values and corresponding *r* values for all 17 protein are then averaged and presented in the row labeled “Ensemble average” in Table 2. This approach decreases the RMSD further compared to using simulated annealing for all but HA (where there is no change) and H (where the RMSD is increased by 0.03 ppm). The effect is largest for N where the RMSD drops by 0.5 ppm and ranges between 0.1 and 0.2 ppm for CA, CB, and C. The *r* values increase by 0.04 to 0.08 for carbon and nitrogen, while no change is observed for HA and a 0.02 decrease is observed for H. To separate the effect of phase space exploration from averaging we use a clustering algorithm to locate the most probable structure in the ensemble, perform a simulated annealing energy minimization starting from this structure, and use the energy minimized structure to compute chemical shifts for each protein. The resulting average RMSD and *r* values are labeled “Annealed ensemble” in Table 2 and suggests that conformational averaging is responsible for most of the 0.4 ppm RMSD reduction observed for N.

In summary, the average RMSD values drop considerably upon minimizing the hybrid energy function using simulated annealing. The largest changes are seen for CA and N, where the RMSD drops by 1.0 and 0.7 ppm on going from the CHARMM structure to the annealed ensemble structure. The drop in RMSD value is also significant for CB (0.4 ppm) and more modest for C (0.3 ppm). For HA and H the drop is also very similar at 0.15 and 0.14 ppm. The difference between the RMSD computed using the annealed CHARMM and ensemble structure is at most 0.2 ppm for carbon and nitrogen.

### Structural changes upon refinement

Table 3 lists the CA-RMSD values of the annealed CHARMM structures (second column) and annealed ensemble structures obtained using ProCS15, CamShift, and force field only relative to the CHARMM minimized structure. The CA-RMSD values of the ProCS15 annealed ensemble structure relative to the ensemble structures are given in parentheses.

**Table 3:** CA RMSD values (in Å) relative to minimized CHARMM structures for annealed CHARMM and annealed ensemble structure obtained using ProCS15, Camshift, and force field only simulations. The values in parenthesis are the CA RMSD deviations of the ProCS15 annealed ensemble structure relative to the ensemble cluster centroid that served as a starting point for the annealing

Protein	Annealed CHARMM ProCS15	Annealed ensemble ProCS15	Annealed ensemble Camshift	Annealed ensemble CHARMM
Maltose-binding periplasmic protein (MPB) (P0AEX9)	0.3	3.9 (0.4)	2.5	6.7
Lin0431 protein (Q92EM7)	0.3	2.9 (0.5)	2.5	7.6
Ubiquitin (P0CG48)	0.5	2.6 (0.7)	2.1	2.6
eh 1 domain from human intersectin-1 (Q15811)	0.3	0.7 (0.4)	1.0	12.3
YbbR family protein (B8FX10)	0.4	1.7 (0.4)	2.2	4.2
Uncharacterized protein from *Chlorobium tepidum* (upCtR107) (Q8KFZ1)	0.3	2.6 (0.5)	2.8	6.8
Methionine sulfoxide reductase (msrB) (P54155)	0.3	3.7 (0.4)	5.7	6.5
26S protease regulatory subunit 8 (P62195)	0.3	1.5 (0.4)	1.2	2.4
drbm 2 domain of interleukin enhancer-b factor 3 (Q12906)	0.5	2.0 (0.7)	3.0	10.5
SMN tudor domain (Q16637)	1.0	1.5 (0.5)	1.4	2.6
Protein G (Q54181)	0.4	1.0 (0.3)	1.0	4.0
Thiamine biosynthesis protein (Q39VC5)	0.4	1.3 (0.5)	0.9	9.1
Lamin-B1 (P20700)	0.5	1.6 (0.4)	1.6	1.9
Target protein XcR50 (Q8P6W3)	0.3	0.6 (0.4)	0.8	2.9
OB-fold domain of replication protein A (Q6LYF9)	0.5	1.5 (0.5)	1.7	2.1
TM1442 protein (Q9X1F5)	0.3	1.5 (0.4)	1.8	1.8
Liver fatty acid-binding protein (LFAB) (P02692)	0.3	2.5 (0.4)	6.5	6.2
Average	0.4	2.0 (0.5)	2.3	5.3

Annealing of the CHARMM structure using ProCS15 changes the CA-RMSD by at most 0.5 Å for all but the SMN tudor domain, where the CA-RMSD is 1.0 Å. The increase in the accuracy of predicted chemical shifts due to annealing the CHARMM structure observed for all the nuclei (*cf.* Table 2) is thus due to very modest changes in the overall structure.

The ensemble calculation followed by simulated annealing results in somewhat larger changes in the overall structure for some of the proteins. For most^11^ of the proteins the overall structural change remains quite modest, with CA-RMSD values ≤2.0 Å relative to the CHARMM minimized structure. For the remaining six proteins (MBP, msrB, Lin0431 protein, ubiquitin, upCtR107, and LFAB) the CA-RMSD range from 2.5 to 3.9 Å. The structural changes are due mostly to the ensemble calculation as the subsequent annealing changes the CA-RMSD by 0.5 Å on average. For five of these six proteins (MBP, msrB, Lin0431 protein, ubiquitin, upCtR107, and LFAB) the structures annealed using only the force field deviate significantly more from the minimized CHARMM structures. So one possible explanation for the relatively large structural changes observed for these proteins is that the force field favors significantly distorted structures and that the inclusion of chemical shifts only partially corrects for these deficiencies in the force field. For comparison, the corresponding annealed ensemble structures using CamShift for msrB and LFAB lead to significantly larger CA-RMSD values (5.7 and 6.5 Å) compared to ProCS, while for MBP the CA-RMSD for CamShift is smaller (2.6 Å). However, for the rest of the proteins the difference in CA-RMSD is generally ≤0.5 Å, except for MBP and the drbm 2 domain of interleukin enhancer-b factor 3 where the difference is 1.4 and 1.0 Å. Next we discuss the six proteins with the largest structural changes in more detail.

#### SMN tudor domain

**Fig. 1:**
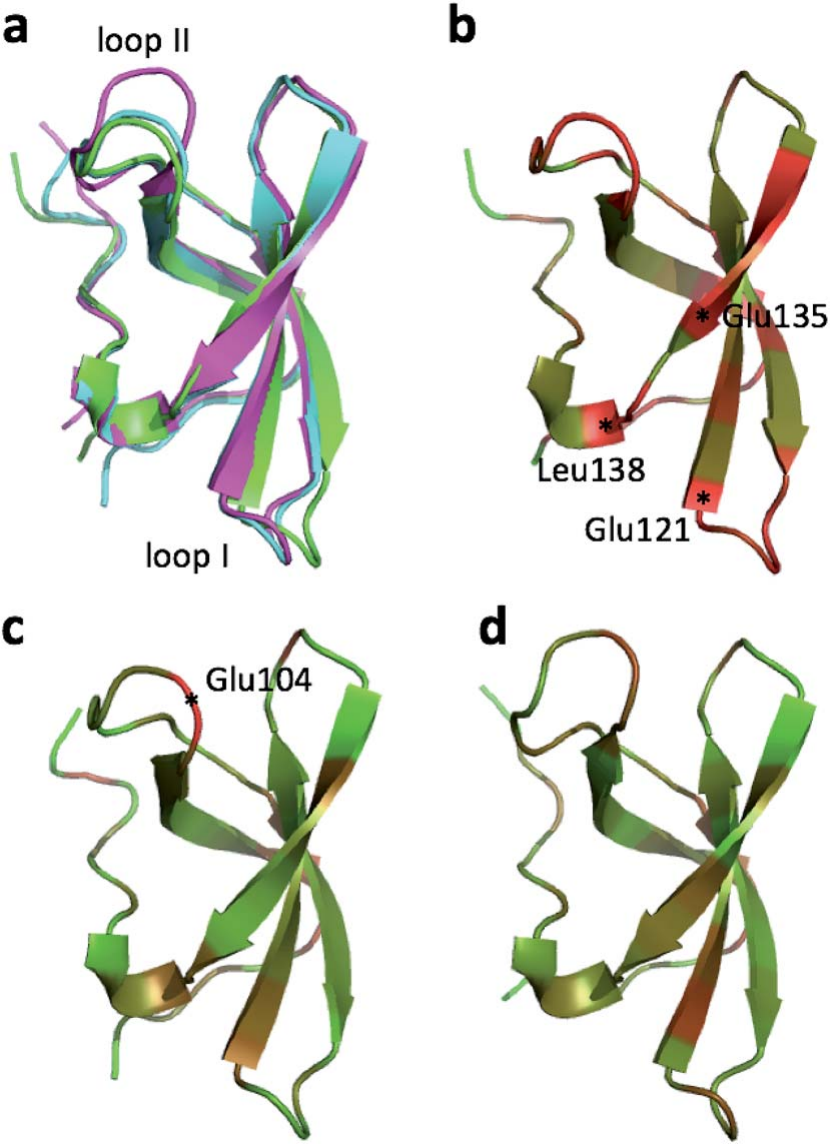
(a) Overlay of CHARMM (green), annealed CHARMM (blue), and annealed ensemble structures (magenta) of the HMN tudor domain. ((b) and (c)) Structure of CHARMM (b), annealed CHARMM (c), and annealed ensemble structures (d) structure colored by *ε* where light green and dark red corresponds to *ε* = 0.0 and *ε* ≥ 2.0, respectively. The structural alignment is made using PyMol.

The annealed CHARMM structure of the SMN tudor domain exhibits the largest CA-RMSD (1.0 Å) from the starting structure (Fig. 1a and Table 3). From Fig. 1a it is evident that most of the structural differences are found at the termini and if the first three and last two residues are neglected the CA-RMSD falls to 0.6 Å. Despite the relatively small structural changes the chemical shifts are improved significantly. For example, the average RMSD values for CA and N chemical shifts decrease from 1.4 to 0.7 ppm and from 3.5 to 2.6 ppm, respectively, when the CHARMM structure is annealed. The changes are quite representative of the corresponding average RMSD values computed for all the proteins (Table 2). Fig. 1b–d shows the CHARMM, annealed CHARMM, and annealed ensemble structures with their colours determined by the chemical shift error (*ε*) computed for each residue14
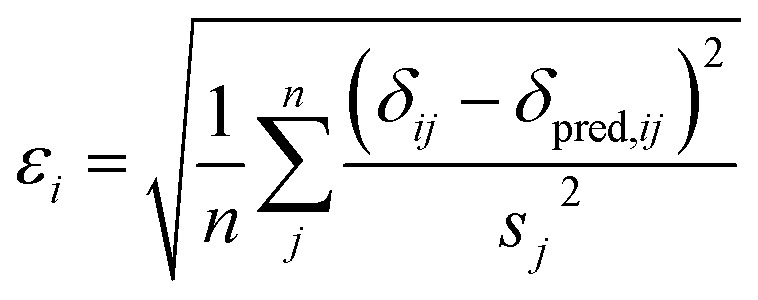
where *δ*_*ij*_ and *δ*_pred,*ij*_ is the experimental and predicted chemical shifts for atom type *j* in residue *i*, *n* is the number of atom types, and the standard deviations (*s*) are taken as the RMSD values computed using the annealed CHARMM structures (Table 2). Note that *ε* is unit-less.

**Fig. 2:**
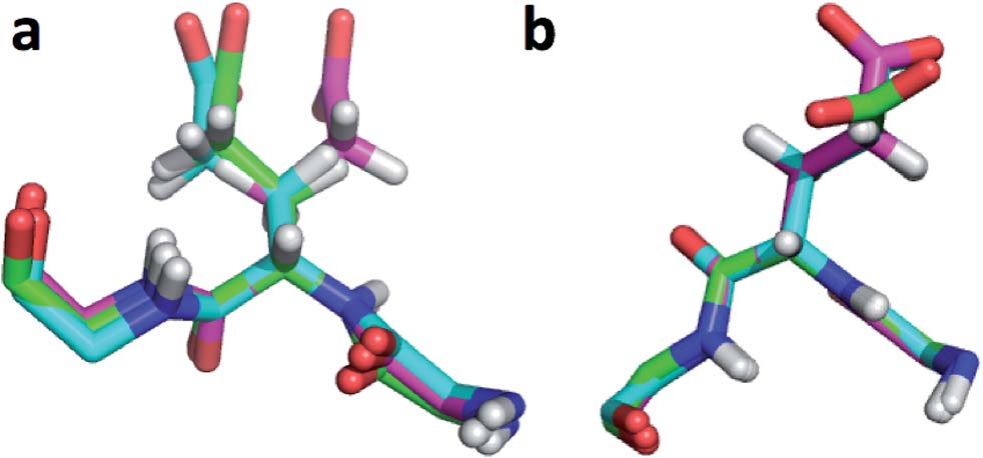
(a) Overlay of CHARMM (green), annealed CHARMM (blue), and annealed ensemble structures (magenta) of the SMN tudor domain in the region around Glu135 (a) and Glu121 (b). The overlay generated in PyMol by minimizing the difference in the position of the C, CA, and N atoms of Glu135 and Glu121, respectively.

The largest overall decrease in error is observed for Glu135, which is primarily due to the fact that the CA and HA error drops from 2.4 to 0.4 ppm and from 0.89 to 0.22 ppm, respectively. Analysis of the chemical shift contributions considered by ProCS15 shows that the chemical shift change is due to changes in the ***φ***/***ψ*** and side-chain dihedral angles (***σ***_BB_ *cf.* ref. 11). Comparison of the CHARMM, annealed CHARMM, and annealed ensemble structure, where the CA and HA errors are 0.6 and 0.16 ppm, indicates that the most likely cause is a 22° change in the ***φ*** angle that alters the interaction with the backbone carbonyl group of Glu136 (Fig. 2a).

The second largest overall error decrease is observed for Glu121 where the error decrease is primarily due to the error for the HA atom decreasing from 1.11 to 0.18 ppm upon annealing. Here the most likely explanation for the decrease in error is a relatively short distance (2.68 Å) between HA and one of the side chain carboxyl atoms in the CHARMM structure, which is increased considerably upon annealing (Fig. 2b). This interaction is also present in the X-ray structure (1HMN, Sprangers *et al.* ^36^) that served as a starting point for the CHARMM minimization, but only in one of the 20 deposited structures determined by NMR (1G5V, Tripsianes *et al.* ^37^).

The largest structural changes upon annealing the CHARMM structure (not counting the termini) is the movement of residues of loop I (Fig. 1a). This loop movement is most likely made to decrease the chemical shift error for Leu138 from 2.0 to 1.2. This error decrease is primarily due to the N chemical shift error changing from 7.3 to 0.9 ppm upon annealing. Analysis of the chemical shift contributions suggest that the cause is the introduction of a NH–O hydrogen bond to the carbonyl oxygen of Glu121. Interestingly, the NH–O is also present in the X-ray structure, *i.e.* the CHARMM/CMAP optimization initiated from the X-ray structure breaks the hydrogen bond, while minimizing the hybrid energy re-forms the hydrogen bond. As a result the loop position in the annealed CHARMM structure is closer to the X-ray structure than to the CHARMM minimized structure.

**Fig. 3:**
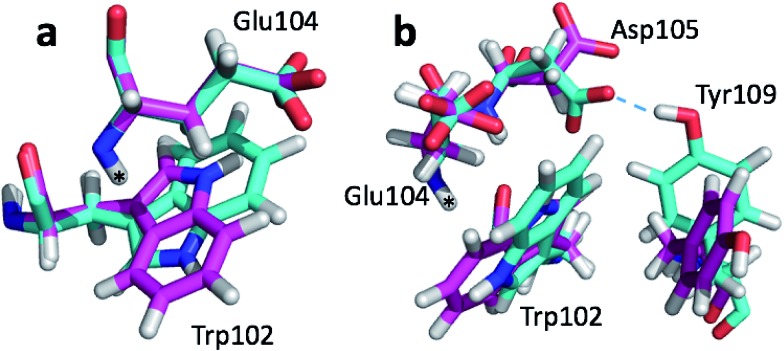
Overlay of annealed CHARMM (blue) and annealed ensemble structures (magenta) of SMN tudor domain in the region around Glu104. The overlay generated in PyMol by minimizing the difference in position of the C, CA, and N atoms of Glu104.

The largest structural differences between the annealed CHARMM and annealed ensemble structure (not counting the termini) is the movement of residues of loop II (Fig. 1a). This loop contains Glu104, which exhibits the largest decrease in error on going from the annealed CHARMM to annealed ensemble structure. This error decrease is primarily due to the H chemical shift error decreasing from 2.04 to 1.16 ppm. Analysis of the chemical shift contributions suggest that the cause is the increase of ring current effect due the Trp102 side chain adopting a different side chain conformation (Fig. 3a). This new side chain conformation leads to the breaking of a hydrogen bond between the Asp105 and Tyr109 side chains which causes the loop movement (Fig. 3b). The Asp105–Tyr109 hydrogen bond is also observed in the X-ray structure but only in one of the 10 NMR ensemble structures. Furthermore, the experimental H chemical shifts of Glu104 and Asp105 are 5.46 and 6.58 ppm, respectively, which makes them the most shielded amide proton chemical shifts observed for this protein – consistent with ring current effects.

To summarize, annealing the CHARMM minimized structure increases the accuracy of the predicted chemical shifts by up to 0.7 and 0.9 ppm for carbon and nitrogen atoms, respectively. The increased accuracy is due to very subtle changes in the protein structure, such as small (22°) changes in a ***φ*** angle, changing side chain conformations, and hydrogen bond formation. A more extensive search of the conformational space lead to slightly more extensive structural changes within a loop involving changes in several side chain conformations and the breaking of a hydrogen bond, that increased ring current effects.

#### Maltose-binding periplasmic protein (MBP)

The largest change in structure upon refinement is observed for MBP with a 3.9 Å CA-RMSD relative to the CHARMM minimized structure (Table 3). For comparison, Lange *et al.* ^38^ used chemical shifts and sparse distance restraints to obtain a structure with an average CA-RMSD of 3.1 Å relative to an X-ray structure (1EZ9,^39^). However, the authors note that the MPB “is a two-domain protein that dynamically samples open and closed conformations in the absence of ligand” and therefore also present average CA-RMSD values of each domain (3.0 and 1.9 Å, for the N-terminal (NTD) and C-terminal domains (CTD)). These values compare reasonably well with corresponding domain RMSD values of 2.0 and 3.1 Å for the annealed ensemble structure relative to the CHARMM minimized structure. This suggests that roughly the same accuracy in domain-structure can be obtained for refinement with and without sparse distance constraints.

**Fig. 4:**
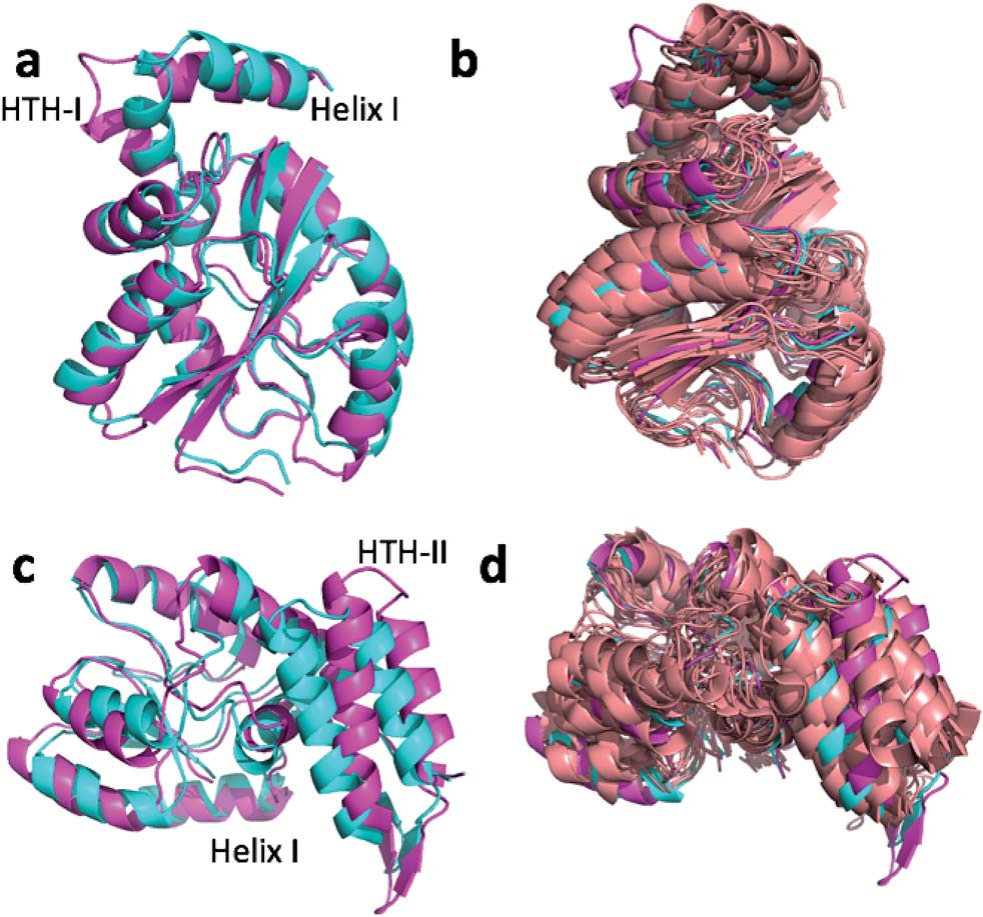
Overlay of annealed CHARMM (blue) and annealed ensemble structures (magenta) of the NTD (a, residues 1–111 and 260–327) and CTD (c, residues 113–258 and 335–370) of MBP. In (c) helix I from (a) is also displayed but not included in the alignment. (b) and (d) include an NMR-derived structural ensemble (2MV0) aligned to the annealed CHARMM structure using PyMol.

Fig. 4a and c show the overlay of the annealed CHARMM and annealed ensemble structure of the NTD and CTD respectively, as defined by Lange *et al.* ^38^ From Fig. 4a it is evident that the largest structural change in the NTD occurs in the helix-turn-helix motif (HTH-I) at the end of the NTD (residues 306–327). The latter helix is in close contact with the CTD, which moves considerably relative to the NTD. In fact, the positions of helices I are much closer to each other when the CTD structures are aligned (Fig. 4a) indicating that, at least in this case, helix I should be included in the CTD domain. The NTD CA-RMSD computed without this helix (residues 311–327) is 1.5 Å, while the corresponding CTD CA-RMSD with this helix included is 3.0 Å. Thus, the comparatively large structural change observed for the HTH-I sub-domain may be driven by inter-domain movement rather than the chemical shifts in the HTH itself. Similarly, Fig. 4c shows that the largest structural change in the CTD occurs in the helix-turn-helix motif (HTH-II) at the end of the CTD (residues 335–370), without which the CA-RMSD drops to 2.3 Å. Fig. 4b and d compare the annealed structures to the NMR ensemble structures (2MV0) obtained by Lange *et al.* ^38^ and indicate that the HTH-II region is quite mobile. In some of the some NMR ensemble structures the position of the loop agrees quite well with the annealed ensemble structures, while others more closely resemble the annealed CHARMM structure.

We note that these large inter-domain and inter-subdomain motions change the chemical shift RMSD values by no more than 0.2 ppm compared to the annealed CHARMM structure so inclusion of the chemical shifts are unlikely to be responsible for these structural changes. Using the Lange *et al.* ^38^ domain definitions the domain CA-RMSD values for the annealed ensemble structure obtained using CamShift are 2.6 and 2.1 for the NTD and CTD respectively and are more similar to the corresponding ProCS15 values than the overall CA-RMSD values listed in Table 3. We note that performing the ensemble calculation without the chemical shifts leads to total and domain CA-RMSD values of 6.8, 4.9 (NTD), and 5.4 Å (CTD), respectively, so chemical shifts are crucial for accurate structures.

#### Methionine sulfoxide reductase (msrB) (P54155)

**Fig. 5:**
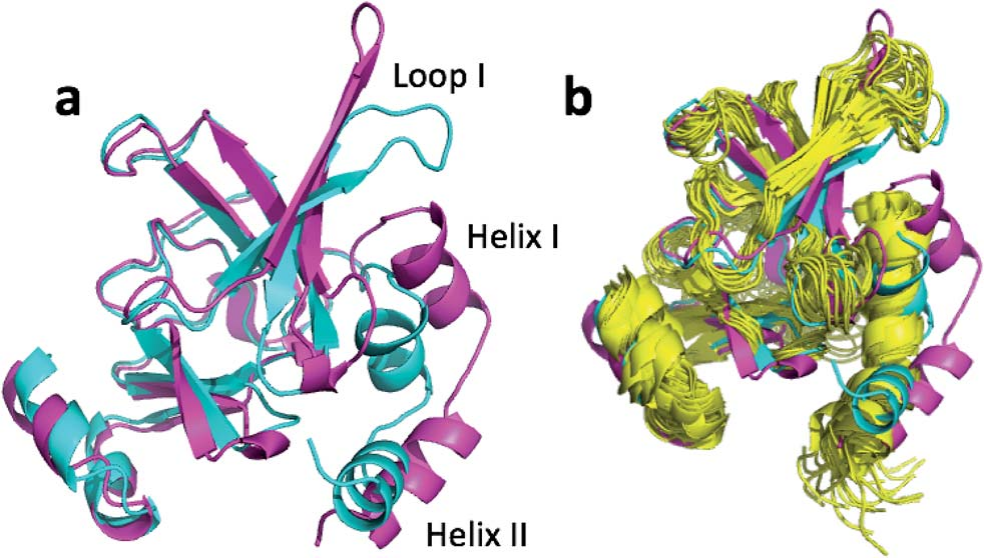
(a) Overlay of annealed CHARMM (blue) and annealed ensemble structures (magenta) of msrB where residues 13–25, 36–105, and 111–141 are used in the alignment. (b) Includes an NMR-derived structural ensemble (3E0O) aligned to the annealed CHARMM structure using PyMol.

The second largest change in structure upon ensemble refinement is observed for msrB with a 3.7 Å CA-RMSD relative to the CHARMM minimized structure (Table 3). If only the structurally ordered parts of the protein, defined by Lange *et al.*,^38^ are used the CA-RMSD drops to 3.2 Å. For comparison, Lange *et al.* ^38^ used chemical shifts, H–N RDCs and sparse distance restraints to obtain a structure with an average CA-RMSD of 1.5 Å relative to an X-ray structure (3E0O, Kim *et al.* ^40^) and conventional NMR leads to a structural ensemble (2KZN, Zheng *et al.* ^41^) with an average RMSD from the X-ray structure of 2.9 Å. Fig. 5 shows the overlay of the annealed CHARMM and annealed ensemble structure and Fig. 5b shows the NMR ensemble structures added as well. From Fig. 5a it is evident that the largest structural change occurs for loop I and helix I and II while Fig. 5b shows that the annealed CHARMM structure and the NMR ensemble structure differ significantly in this region as well. The positions of loop 1 and the top of helix II in the annealed ensemble structure are arguably in better agreement with the NMR ensemble structures than the annealed CHARMM structure despite the fact that the NMR ensemble is aligned to the annealed CHARMM structure in the figure. However, the structural variability of loop I makes it difficult to quantify this agreement *via* CA-RMSD.

The ensemble structure annealed using CamShift has a CA-RMSD that is 2.1 Å higher than the ensemble structure annealed using ProCS15 (Table 3). However, much of the structural discrepancy occurs around loop I and helix I and II making it difficult to argue that the annealed CamShift ensemble structure is necessarily of worse quality.

#### Lin0431 protein (Q92EM7)

**Fig. 6:**
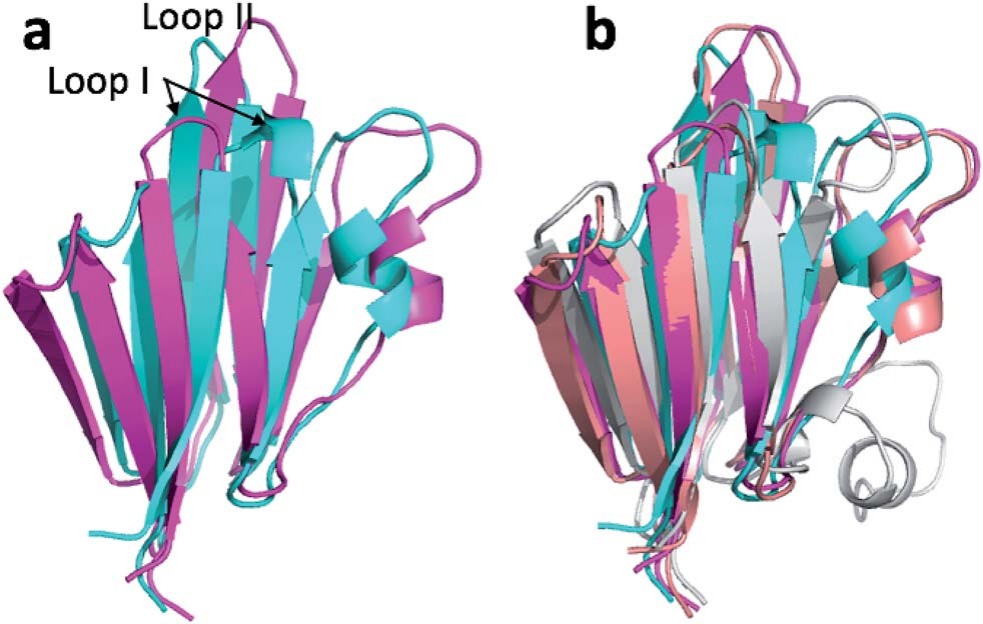
(a) Overlay of annealed CHARMM (blue) and annealed ensemble structures (magenta) of Lin0431. (b) Includes the annealed CamShift (beige) and CHARMM (grey) ensemble structures.

The third largest change in structure upon ensemble refinement is observed for msrB with a 2.9 Å CA-RMSD relative to the CHARMM minimized structure (Table 3). Fig. 6a shows the overlay of the annealed CHARMM and annealed ensemble structure and reveals that the change in structure is an interdomain movement between domains consisting of four beta sheets (domain I) and three beta sheets plus a short alpha helix (domain II). The interdomain movement is very similar for corresponding simulations with CamShift (Fig. 6b) but significantly more pronounced when no chemical shifts is used in the refinement. The most likely explanation for the large domain movement is therefore deficiencies in the underlying force field that the inclusion of the chemical shifts is not able to counteract completely.

#### Ubiquitin (P0CG48)

The fourth largest change in structure (tied with the following two proteins) upon ensemble refinement is observed for ubiquitin with a 2.6 Å CA-RMSD relative to the CHARMM minimized structure (Table 3). Inspection of the structure shows that the change in structure is primarily in the C-terminal tail and if the last five residues are excluded the CA-RMSD drops to 0.9 Å.

#### Uncharacterized protein from *Chlorobium tepidum* CtR107 (upCtR107) (Q8KFZ1)

**Fig. 7:**
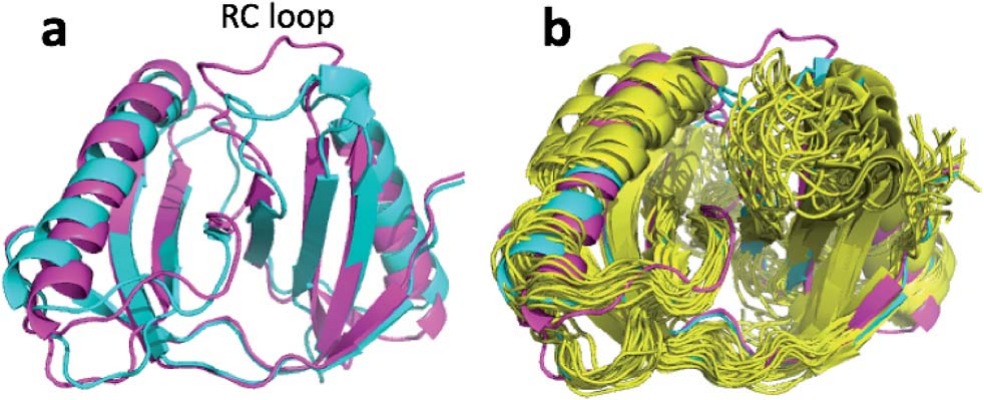
(a) Overlay of annealed CHARMM (blue) and annealed ensemble structures (magenta) of upCtR107. (b) Includes the NMR-derived structural ensemble 2KCU aligned to the annealed CHARMM structure using PyMol.

A similarly large change in structure upon ensemble refinement is observed for upCtR107 with a 2.6 Å CA-RMSD relative to the CHARMM minimized structure (Table 3). Fig. 7a shows the overlay of the annealed CHARMM and annealed ensemble structure and reveals that the change in structure is primarily in a random coil loop (RC loop in figure) connecting two beta strands while Fig. 7b shows that the annealed CHARMM structure and the NMR ensemble structure (2KCU) differ significantly in this region as well. Unlike msrB the annealed ensemble structure cannot be said to be in better agreement in the NMR ensemble but the disorder in this region of NMR ensemble is so large that a statement regarding the quality of the structure in this region is not really meaningful. If the loop region (residues 131–143) is excluded from the CA-RMSD calculation then the CA-RMSD drops to 1.8 Å.

#### Liver fatty acid-binding protein (LFABP) (P02692)

**Fig. 8:**
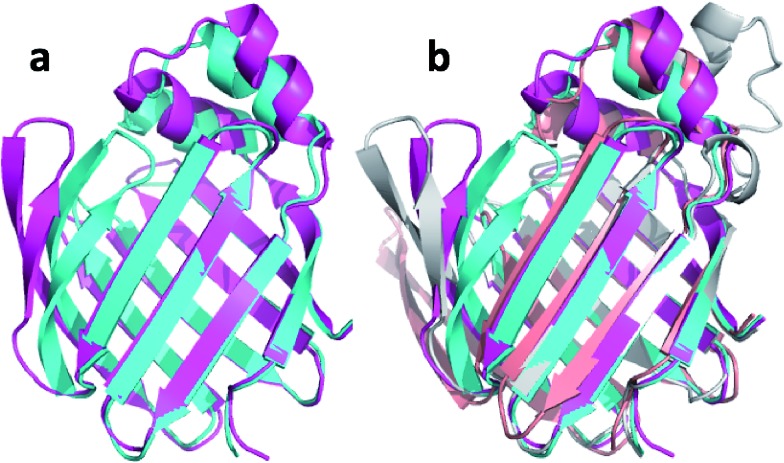
(a) Overlay of annealed CHARMM (blue) and annealed ensemble structures (magenta) of Lin0431. (b) Includes the annealed CamShift (beige) and CHARMM (grey) ensemble structures.

The final protein in Table 3 to exhibit a >2.0 Å change in structure upon ensemble refinement (2.6 Å CA-RMSD) is LFABP. Fig. 8a shows the overlay of the annealed CHARMM and annealed ensemble structure and reveals that the change in structure is primarily in the two alpha helices and a beta-hairpin. Intriguingly, it is also these two regions that move during ligand binding (Fig. S1†) when the protein goes from an open to a closed form, so these are known to be flexible regions of the protein. The X-ray structure used as the starting point for the simulations is of the closed ligand-bound form while the chemical shifts are those measured in the absence of the ligand, which could explain why the annealed CHARMM structure is more open. However, performing the same simulation with chemical shifts measured for the ligand bound form resulted in an annealed ensemble structure that was virtually identical to the one shown in Fig. 8a (with an CA-RMSD of only 0.3 Å).

The movements of the beta hairpin and alpha helices are very similar for corresponding simulations with CamShift and CHARMM-only (Fig. 8b) but, in the case of the beta hairpin, significantly more pronounced when no chemical shifts are used in the refinement. The most likely explanation for the high CA-RMSD for the annealed ensemble structure is therefore deficiencies in the underlying force field that the inclusion of the chemical shifts is not able to counteract completely. LFABP is thus the only instance where ProCS15 seems to provide a structure that is significantly closer to the experimental structure than CamShift.

In summary, there are six proteins for which the CA-RMSD of the annealed ensemble structures differ by >2 Å from the starting CHARMM structure. For four of the proteins the large structural change is either due to domain or sub-domain motion (MPB) or loop/tail movement in regions of the protein that are disordered in the corresponding NMR ensembles (msrB, ubiquitin, and upCtR107) and CA-RMSD values computed for domains or excluding disordered regions range from 0.9 to 2.3 Å. For the remaining two proteins (Lin0431 and LFABP) the most likely explanation for the large structural change is deficiencies in the force field that inclusion of the chemical shifts only partly ameliorate. Despite the large structural changes, the predicted chemical shift RMSD values change by, on average, 0.1 ppm and 0.01 ppm for carbon/nitrogen and hydrogen, respectively.

### Accuracy of chemical shift predictors using refined structures

Before we compare the accuracy of ProCS15 predictions computed using the annealed structures with the empirical methods we note that the results can be improved further for some nuclei by introducing a separate chemical shift offset for each type of amino acid. The offset for a given residue is the average deviation from experiments for the specific amino acid type averaged for each of the 17 proteins, with the values given in Table S3.† A similar correction is also done in CheShift-2 (ref. 9) and SHIFTS.^6^ Furthermore, the empirical methods all compute chemical shifts as structure-dependent corrections to random coil values for each amino acid type, so this correction is also implicitly included in these methods.

Table 4 lists the average RMSD and *r* values computed for all 17 proteins using the CHARMM, annealed CHARMM, ensemble, and annealed ensemble structures. Comparison to the corresponding values in Table 2 shows that the amino acid type specific correction lowers the average RMSD by 0.0 to 0.3 ppm for carbon and nitrogen while it has a negligible effect on the hydrogen chemical shifts. The effect tends to be largest (0.2–0.3 ppm) for CB and smallest for (0.0–0.1 ppm) for CA.

**Table 4:** Average RMSD (in ppm) and *r* values relative to experiment of chemical shifts computed using ProCS15 with amino acid specific corrections for 17 different proteins and various structural optimization techniques

	CA	CB	C	HA	H	N
CHARMM	1.5 (0.73)	1.6 (0.62)	1.5 (0.50)	0.30 (0.78)	0.54 (0.52)	3.2 (0.65)
Annealed CHARMM	0.8 (0.91)	1.3 (0.76)	1.4 (0.58)	0.18 (0.92)	0.39 (0.79)	2.6 (0.76)
Ensemble average	0.6 (0.95)	1.1 (0.83)	1.3 (0.64)	0.17 (0.93)	0.43 (0.76)	2.2 (0.83)
Annealed ensemble	0.6 (0.95)	1.3 (0.76)	1.4 (0.62)	0.17 (0.93)	0.40 (0.78)	2.4 (0.79)

Table 5 lists the average RMSD and *r* values computed using annealed CHARMM structures using ProCS15 with amino acid type specific corrections, CheShift-2, and five popular empirical chemical shift predictors. We choose to use annealed CHARMM structures rather than annealed ensemble structures because the former more closely match the X-ray structures and many of the empirical predictors are parameterized using X-ray structures. The ProCS15 error is now lower than for CamShift for all atom types except CB and C. For CA, H, and HA atoms the average RMSD and *r* values are now comparable to PPM_One, Sparta+, and shAIC, while the average RMSD values predicted by ShiftX2 for CA and H are still 0.3 and 0.11 ppm lower than for ProCS15. In the case of C, the average RMSD is still 0.2–0.7 ppm higher than for the empirical methods, which may be due to the double zeta basis set (OPBE/6-31G(d,p)) used to parameterize ProCS15.^42^ In the case of CB and N, the average RMSD values are 0.1–0.4 ppm and 0.4–0.5 ppm higher than for PPM_One, Sparta+, and shAIC, and 0.6 and 1.0 ppm higher than is ShiftX2. However, the average RMSD values for these nuclei can be decreased by 0.2 and 0.4 ppm, respectively, by averaging over many structures (Table 4), which makes the average RMSD values quite comparable to the empirical methods, with the exception of ShiftX2.

**Table 5:** Computed using annealed CHARMM structures. ProCS15 results use amino acid type specific correction

	CA	CB	C	HA	H	N
ProCS15	0.8 (0.91)	1.3 (0.76)	1.4 (0.58)	0.18 (0.92)	0.39 (0.79)	2.6 (0.76)
CheShift-2	1.3 (0.76)	1.6 (0.54)				
CamShift	1.0 (0.83)	1.2 (0.74)	1.2 (0.73)	0.24 (0.86)	0.47 (0.72)	2.9 (0.65)
PPM_One	0.7 (0.93)	0.9 (0.86)	1.0 (0.81)	0.19 (0.92)	0.36 (0.83)	2.1 (0.82)
Sparta+	0.7 (0.92)	0.9 (0.86)	0.9 (0.83)	0.21 (0.90)	0.37 (0.82)	2.1 (0.83)
shAIC	0.7 (0.91)	0.9 (0.84)	0.9 (0.85)	0.20 (0.90)	0.41 (0.78)	2.2 (0.82)
ShiftX2	0.5 (0.94)	0.7 (0.90)	0.7 (0.90)	0.13 (0.94)	0.28 (0.88)	1.6 (0.87)

Comparison of the average RMSD values in Tables 4 and 1 show that the accuracy of CheShift-2 and the empirical methods are slightly improved by annealing the CHARMM structure with ProCS15 for some methods and some nuclei. The improvements, if any, are usually 0.1 ppm for carbon and nitrogen atoms and up to <0.05 ppm for hydrogen atoms. Though modest, the overall RMSD lowering may suggest that the minor structural changes introduced by ProCS15-based annealing improves the accuracy of the protein structures.

## Summary and outlook

We present a method by which quantum chemistry-based predictions of isotropic chemical shielding values (ProCS15) can be used to refine protein structures using Markov Chain Monte Carlo (MCMC) simulations and a hybrid energy function based in a standard force field (CHARMM/CMAP) weighted by the agreement energy between computed chemical shielding values and measured chemical shifts. The slope and intercept relating the chemical shielding values to the experimental chemical shifts are included probabilistically, together with the weights as previously reported.^19^ Two kinds of MCMC structural refinement simulations were performed using CHARMM/CMAP geometry optimized X-ray structures as starting points: simulated annealing of the starting structure or constant temperature MCMC simulation followed by simulated annealing of a representative ensemble structure.

As we observed previously (Larsen *et al.*, 2015) the chemical shift RMSDs from experiment for ProCS15 are significantly higher (0.5–1.4 ppm for carbon and N atoms) than those for commonly used empirical chemical shift predictors and very similar to CheShift values, while the corresponding ProCS15 *r* values are lower than for the empirical methods and similar to CheShift-2. However, we show that the average RMSD values drop considerably upon minimizing the hybrid energy function using simulated annealing. The largest changes are seen for CA and N, where the RMSD drops by 1.0 and 0.7 ppm on going from the CHARMM structure to the annealed ensemble structure. The drop in RMSD value is also significant for CB (0.4 ppm) and more modest for C (0.3 ppm). For HA and H the drop is also very similar at 0.15 and 0.14 ppm. Annealing of the CHARMM structure using ProCS15 changes the CA-RMSD by at most 0.5 Å for all but SMN tudor domain, where the CA-RMSD is 1.0 Å. The increase in the accuracy of the predicted chemical shifts due to annealing the CHARMM structure observed for all the nuclei is thus due to very modest changes in the overall structure.

In order to explore an even larger region of phase space and the effect of conformational averaging we perform a constant temperature Monte Carlo simulation for each protein using a hybrid energy function. There are six proteins for which the CA-RMSD of the annealed ensemble structures differ by >2 Å from the starting CHARMM structure. For four of the proteins the large structural change is either due to domain or sub-domain motion (MPB) or loop/tail movement in regions of the protein that are disordered in the corresponding NMR ensembles (msrB, ubiquitin, and upCtR107) and CA-RMSD values computed for domains or excluding disordered regions range from 0.9 to 2.3 Å. For the remaining two proteins (Lin0431 and LFABP) the most likely explanation for the large structural change is deficiencies in the force field that inclusion of the chemical shifts only partly ameliorate. Despite the large structural changes the predicted chemical shift RMSD values change by, on average, 0.1 ppm and 0.01 ppm for carbon/nitrogen and hydrogen, respectively. Annealed ensemble structures obtained using CamShift have CA-RMSD values that are within 0.5 Å of the corresponding ProCS15 values for all but three structures, where the ProCS15 CA-RMSD values are lower by 0.8, 2.0, and 4.0 Å.

The accuracy of the ProCS15 chemical shifts can be improved by introducing an chemical shift offset for each amino acid type, which lowers the average RMSD by 0.0 to 0.3 ppm for carbon and nitrogen while it has a negligible effect on the hydrogen chemical shifts. For CA, H, and HA atoms the average RMSD and *r* values computed using a single structure are now comparable to the empirical predictors. This is also the case for CB and N, if dynamical averaging is included, while for C the RMSD values remain 0.1–0.6 ppm higher – most likely due to basis set effects. The overall accuracy of CheShift-2 and the empirical methods are slightly improved by annealing the CHARMM structure with ProCS15, which may suggest that the minor structural changes introduced by ProCS15-based annealing improves the accuracy of the protein structures.

Having established that QM-based chemical shift prediction can deliver the same accuracy as empirical shift predictors we hope this can help increase the accuracy of related approaches such as QM/MM or linear scaling approaches^12–15^ or interpreting protein structural dynamics from QM-derived chemical shifts.^43^ For example, in former case it will be interesting to see if the use of ProCS15 annealed structures lead to better predictions and/or whether ProCS15 can be used to identify suitable MD snapshots.^44^ In addition further work on ProCS15 is needed to increase the accuracy of the underlying DFT calculations (especially for C) and the methods used to interpolate between grid points in the DFT database, as well as extending the approach to the prediction chemical shifts of additional atoms in the side chains. Work in these areas is currently underway.
