# Supplementary material for: Protein structure refinement using a quantum mechanics-based chemical shielding predictor
Source: Chem Sci. 2016 Dec 1;8(3):2061–72. doi: 10.1039/c6sc04344e (PMC5399634; doi:10.1039/c6sc04344e)
Supplement: Supplementary file 1 [file SC-008-C6SC04344E-s001.pdf]

Electronic Supplementary Material (ESI) for Chemical Science.
This journal is © The Royal Society of Chemistry 2016

SUPPORTING INFORMATION

Additional supplementary material, including the structures used in the chemical shift prediction, can be found on Figshare: dx.doi.org/10.6084/m9.figshare.3856416

Table S1. UniProt codes, names, PDB IDs and resolution of the X-ray structures, residues included in the models, and the BMRB codes of the chemical shift data of the 17 proteins used in this study. ”a” indicates an NMR refined x-ray structure.

UniProt	Name	PDB ID	Resolution	residues	BMRB
B8FX10	YbbR family protein	3LYW	1.9	37-119	16570
P02692	Fatty acid-binding protein, liver	1LFO	2.3	1-127	15429
P0AEX9	Maltose-binding periplasmic protein	1LLS	1.8	27-396	25237
P0CG48	Ubiquitin	1UBQ	1.8	1-76	17919
P20700	Lamin-B1	3UMN	2	428-550	16572
P54155	Methionine sulfoxide reductase MsrB	3E0O	2.6	1-143	17008
P62195	26S protease regulatory subunit 8	3KW6	2.1	318-395	16640
Q12906	drbm 2 domain of interleukin enhancer-b factor 3	3P1X	1.9	520-594	17169
Q15811	eh 1 domain from human intersectin-1	3FIA	1.45	1-111	16250
Q16637	SMN Tudor Domain	1MHN	1.8	89-147	18005
Q39VC5	thiamine biosynthesis protein (ThiS)	3CWI	1.9	1-70	15844
Q54181	GB3	2OED	a	132-185	1639
Q6LYF9	OB-fold domain of replication protein A	3E0E	1.6	173-267	15849
Q8KFZ1	uncharacterized protein from Chlorobium tepidum CtR107	3E0H	1.81	1-158	16097
Q8P6W3	target protein XcR50	1TTZ	2.11	1-78	6363
Q92EM7	Lin0431 protein	3LD7	1.55	36-127	16563
Q9X1F5	TM1442 protein	1VC1	2	1-110	5921

Table S2. Modifications to X-ray structures.

PDB-id	Comment
3LYW (B8FX10)	Added missing side-chain to GLU-42<br>Removed C-terminal residues 84-86
1LLS (P0AEX9)	ARG-230 mutated to TRP
3KW6 (P62195)	Added missing side-chain to GLN-73
3P1X (Q12906)	Removed residue GLY-525<br>Added missing side-chain to<br>LYS-526, ARG-537, LYS-540, GLU-542, SER-550, HIS-551, ASP-552, LYS-553
3FIA (Q15811)	Removed N-terminal residues 6-12
3E0E (Q6LYF9)	THR-21 mutated to ALA-22<br>Added missing residues 30-33, 79, 80<br>Added missing side-chains to THR-34, LYS-35, LYS-76, GLN-77<br>Added C-terminal GLU-97
3E0H (Q8KFZ1)	THR-27 mutated to ALA-27
3LD7 (Q92EM7)	Removed N-terminal GLY-39

Table S3. Amino acid specific offsets for each atom type

	CA	CB	C	HA	H	N
CYS	-0.50	2.70	1.40	-0.03	0.05	0.75
GLN	-0.25	1.20	-0.55	0.01	-0.05	-1.00
ILE	-0.03	0.05	0.00	0.01	-0.05	-0.40
SER	-0.10	0.85	0.50	-0.11	0.00	1.20
VAL	-0.30	1.10	0.30	0.00	0.00	0.10
LYS	0.05	0.30	-0.75	0.05	0.00	-0.70
PRO	-0.05	-0.40	-0.50	-0.09	0.00	0.00
GLY	-0.10	0.00	0.60	-0.25	-0.15	1.75
THR	-0.15	-0.20	0.55	-0.01	0.15	1.80
PHE	-0.30	-0.50	0.20	-0.04	-0.05	0.15
ALA	0.25	-0.90	-1.10	0.00	0.15	-1.35
HIS	-0.15	1.20	0.15	-0.05	0.00	0.30
MET	-0.05	-0.10	0.00	0.00	0.05	-0.60
ASP	0.65	-1.40	0.95	0.13	0.05	0.85
GLU	0.40	1.40	-0.10	0.01	0.05	-0.15
LEU	-0.10	-1.00	-0.05	0.16	-0.05	-0.55
ARG	0.00	0.20	-0.60	0.00	-0.15	-0.55
TRP	-0.30	0.50	-1.20	0.05	-0.05	-1.55
ASN	0.36	-0.80	0.00	-0.10	-0.05	0.15
TYR	-0.15	-0.20	0.40	0.00	-0.10	-0.05

Table S4. Average slopes computed using ProCS15 for 17 different proteins and various structural refinement techniques

	CA	CB	C	HA	H	N
CHARMM	-1.05	-0.94	-0.39	-0.51	-0.32	-0.51
Annealed CHARMM	-1.10	-0.95	-0.49	-0.64	-0.56	-0.58
Ensemble average	-1.11	-0.96	-0.36	-0.64	-0.62	-0.68
Annealed ensemble	-1.12	-0.96	-0.54	-0.65	-0.54	-0.60

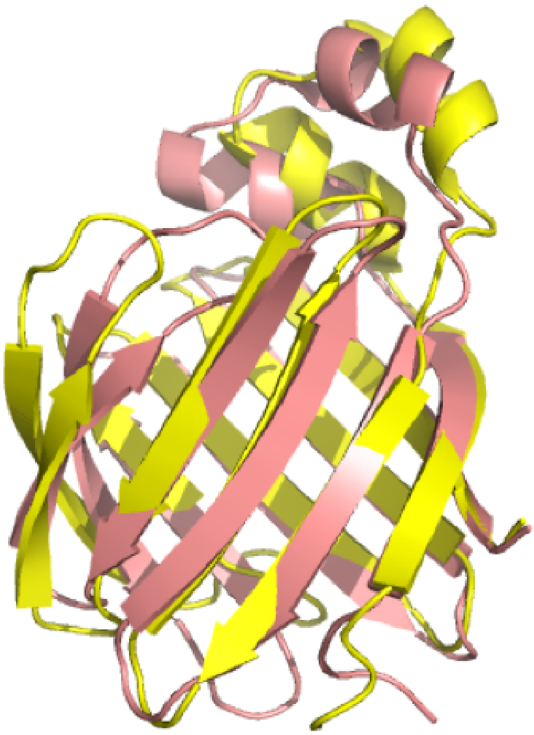

Figure S1. Overlay of two representative NMR structures obtained for the apo- (2JU3, yellow) and holo-form (2JU7, beige) of LFABP.
